# Digital Interventions to Support Population Mental Health in Canada During the COVID-19 Pandemic: Rapid Review

**DOI:** 10.2196/26550

**Published:** 2021-03-02

**Authors:** Gillian Strudwick, Sanjeev Sockalingam, Iman Kassam, Lydia Sequeira, Sarah Bonato, Alaa Youssef, Rohan Mehta, Nadia Green, Branka Agic, Sophie Soklaridis, Danielle Impey, David Wiljer, Allison Crawford

**Affiliations:** 1 Centre for Addiction and Mental Health Toronto, ON Canada; 2 University of Toronto Toronto, ON Canada; 3 University Health Network Toronto, ON Canada; 4 University of Alberta Edmonton, AB Canada; 5 Mental Health Commission of Canada Ottawa, ON Canada

**Keywords:** digital health, psychiatry, mental health, informatics, pandemic, COVID-19, telemedicine, eHealth, public health, virtual care, mobile apps, population health

## Abstract

**Background:**

The COVID-19 pandemic has resulted in a number of negative health related consequences, including impacts on mental health. More than 22% of Canadians reported that they had felt depressed in the last week, in response to a December 2020 national survey. Given the need to physically distance during the pandemic, and the increase in demand for mental health services, digital interventions that support mental health and wellness may be beneficial.

**Objective:**

The purpose of this research was to identify digital interventions that could be used to support the mental health of the Canadian general population during the COVID-19 pandemic. The objectives were to identify (1) the populations these interventions were developed for, inclusive of exploring areas of equity such as socioeconomic status, sex/gender, race/ethnicity and culture, and relevance to Indigenous peoples and communities; (2) the effect of the interventions; and (3) any barriers or facilitators to the use of the intervention.

**Methods:**

This study was completed using a Cochrane Rapid Review methodology. A search of Embase, PsycInfo, Medline, and Web of Science, along with Google, Million Short, and popular mobile app libraries, was conducted. Two screeners were involved in applying inclusion criteria using Covidence software. Academic articles and mobile apps identified were screened using the Standard Quality Assessment Criteria for Evaluating Primary Research Papers from a Variety of Fields resource, the American Psychiatric Association App Evaluation Framework, and the Mental Health Commission of Canada’s guidance on app assessment and selection.

**Results:**

A total of 31 mobile apps and 114 web-based resources (eg, telemedicine, virtual peer support groups, discussion forums, etc) that could be used to support the mental health of the Canadian population during the pandemic were identified. These resources have been listed on a publicly available website along with search tags that may help an individual make a suitable selection. Variability exists in the populations that the interventions were developed for, and little assessment has been done with regard to areas of equity. The effect of the interventions was not reported for all those identified in this synthesis; however, for those that did report the effect, it was shown that they were effective in the context that they were used. A number of barriers and facilitators to using these interventions were identified, such as access, cost, and connectivity.

**Conclusions:**

A number of digital interventions that could support population mental health in Canada during the global COVID-19 pandemic were identified, indicating that individuals have several options to choose from. These interventions vary in their purpose, approach, design, cost, and targeted user group. While some research and digital interventions addressed equity-related considerations, more research and focused attention should be given to this area.

## Introduction

### Background

Prior to the onset of the COVID-19 pandemic, 20% of Canadians were estimated to experience a mental illness in any given year [1]. In addition, 1 in 3 Canadians were expected to experience a mental illness during their lifetime [2]. The probability of experiencing mental illness or having suboptimal mental health was found to be significantly more likely for Canadians within socially and economically marginalized populations [3]. As a result of the COVID-19 pandemic, and the uncertainty, fear, and stress created during this unprecedented time, concern has been raised both in the media and academic literature regarding the emerging and concerning mental health crisis within Canada [4]. The economic hardship, isolation, and social distancing measures enacted to slow the virus spread have inadvertently introduced a number of mental health consequences, including increased feelings of anxiety, depression, distress, insomnia, and burnout [5]. The decline in population mental health appears to be a direct result of the worsening public health crisis, which has been detrimental to both Canadian and international populations.

Data from several countries have identified increased risk and burden of mental health distress during COVID-19. A recent survey from the United States found that the prevalence of depression symptoms increased more than 3-fold during the pandemic [6]. Prior to the pandemic, 8.5% of individuals in the United States expressed feelings of depression; this number increased to 27.8% during the COVID-19 pandemic [6]. The study also found that individuals in lower-income brackets were 1.5 times more likely to experience symptoms of depression in comparison to those in higher-income groups [6]. Furthermore, a survey conducted by the Centers for Disease Control and Prevention of US citizens concluded that the adverse mental health impacts created by the COVID-19 pandemic have disproportionately affected marginalized populations, specifically those who identify as persons of color, younger adults, caregivers, and those with pre-existing mental health conditions [7]. Similarly, symptoms of depression, anxiety, and stress increased during the first 4-6 weeks of social distancing and isolation measures in the United Kingdom [8]. The substantial decline in mental well-being in the United Kingdom shows a comparable pattern to the United States, with disproportionate and inequitable impact on marginalized populations. There is a significantly higher prevalence of mental distress among individuals who have pre-existing physical and mental health conditions, those who identify as persons of color, and those in lower-income brackets [9]. The international mental health effects of the COVID-19 pandemic are also evident in the Canadian population [10].

A recent survey conducted by the Centre for Addiction and Mental Health (CAMH), in December 2020, found that 24% of Canadians experienced moderate to severe anxiety, 23% felt lonely, and 22% felt depressed as a result of COVID-19 [11]. Women experienced worse mental health outcomes in comparison to men, where 25% of women reported feeling more depressed, anxious, and lonely during the pandemic period [12]. Moreover, thoughts and feelings of suicide in the Canadian population have notably risen since the onset of COVID-19, whereby 6% of the Canadian population experienced thoughts or feelings of suicide [13]. In comparison to the general Canadian population, Indigenous peoples and communities, individuals with pre-existing mental health conditions, and individuals in lower-income brackets were 2 to 4 times more likely to have thoughts or feelings of suicide [13]. The lasting impact of the COVID-19 pandemic on the mental health and well-being of Canadians warrants concern as individuals, primarily vulnerable and marginalized populations, are already at greater risk of developing severe and chronic mental health disorders [14].

Stressors created by enforced social distancing and isolation measures have applied further pressure on an already overburdened, strained, and limited Canadian mental health care system [10]. Despite the recognizable need for increased mental health supports, available mental health services are not adequate to support this increased volume of need through conventional services such as face-to-face care [15,16]. In addition, due to constraints posed by COVID-19, it is no longer feasible or appropriate to provide in-person mental health care services under many circumstances [15,16]. Where face-to-face interventions are offered, these currently address acute and severe decompensation of mental health. There is a need to support the adaptation, transformation, and augmentation of our current infrastructure to increase capacity and ideally to support mental health and wellness at a population health level [10].

Digital interventions created in the response to COVID-19 are potential solutions to support population mental health during and after the pandemic. By digital interventions, we refer to those in the World Health Organization’s (WHO) classification of digital health technologies [17], as well as the types outlined by the Mental Health Commission of Canada (MHCC) [18] such as websites, web-based programs, electronic knowledge platforms, mobile health apps (inclusive of texting), telemedicine, and social media. Digital interventions enable the provision of convenient and timely mental health care services and support through digital means. Digital interventions that have been created for non–COVID-19–related purposes (eg, for natural and human-made disasters, other pandemics) may also be relevant to support population mental health. This approach has been utilized for the identification of nondigital interventions during the COVID-19 pandemic [19].

### Research Question and Objectives

The following research question was asked: what digital interventions could be used to support the mental health of the Canadian general population during the COVID-19 pandemic?

The objectives of the study were the following:

1. To identify populations that digital interventions might impact. We are specifically interested in understanding key equity areas related to sex/gender, socioeconomic status, race/ethnicity and culture, and relevance to Indigenous peoples and communities;
2. To determine what is known about the effect of these digital interventions and for whom;
3. To identify barriers and facilitators to the use of these digital interventions.

## Methods

We conducted a rapid review of the academic and gray literature using the interim guidance from the Cochrane Rapid Reviews Methods Group with minor modifications made where appropriate [20].

### Search Strategy

For the academic searches, our librarian team member created detailed search strategies and executed searches for Embase, PsycInfo, Medline, and Web of Science. We have included the preliminary search strategy for Medline in Multimedia Appendix 1. Search strategies for other databases were developed based on the Medline search strategy approach. For the gray literature review, we conducted searches of smartphone app stores, curated smartphone app libraries, and structured Google searches [21]. We conducted a brainstorming session with 3 members of the investigator team, inclusive of a person with lived experience of mental health problems or illnesses to develop a list of key terms the public may search when looking for smartphone apps to support their mental health during COVID-19. The list of key terms was then presented to and approved by the larger study team. The Google Play Store and Apple’s App Store were searched using the terms identified during the brainstorming session. The top 10 smartphone apps per search term were included in this review.

We searched the following curated smartphone libraries: Practical Apps, Alberta Health Services App Library, Scarborough Health Network Mental Health App Library, eMentalhealth.ca, King’s Western University Library, Health Navigator New Zealand, NHS App Library, and One Mind Psyberguide. A gray literature search on Google using a structured Google search was also completed (search string: COVID + Mental Health + apps). The structured Google search methodology aimed to identify information from a diverse range of locations to account for the personalization of Google search results [22]. Our librarian team member conducted structured searches of Google and Million Short search engines to identify web-based resources and/or smartphone apps that may support population mental health during COVID-19. Million Short, a Canadian web search engine, was used in addition to Google, as it identifies websites that would otherwise not be highly ranked in other search engine results [23]. We reviewed the first 30 search findings (unique websites) from the 62 searches completed using the Google Chrome web browser. Paid ads were not present within the searches completed. A complete list of Google and Million Short search strings are available in Multimedia Appendix 2.

### Inclusion Criteria

The following inclusion criteria were utilized for academic searches:

- Academic articles must describe a digital mental health intervention;
- Digital interventions are classified based on both the MHCC technology types [18], and the WHO classification of digital interventions [17]. The MHCC and WHO classifications were mapped to one another. Table 1 presents the mapped breakdown of digital health intervention types;
- Published since 2000;
- Written in English;
- Described a population mental health treatment, assessment, promotion, or prevention intervention, inclusive of promoting mental well-being and delivering mental health services;
- Relevant to the COVID-19 context (eg, natural disasters, man-made disasters, other medical pandemics, etc) [19]. Web-based resources that were COVID-19 specific were included;
- Research articles, commentaries, reviews, and nonresearch articles were included.

The following inclusion criteria were utilized for gray literature searches:

- Available for use within Canada by the general public;
- Developed specifically for or modified for the COVID-19 pandemic. Resources that were modified for the COVID-19 pandemic include:
  - Resources that have been suggested as a mental wellness resource during the pandemic;
  - Resources that have been updated during COVID-19 to include relevant mental health resources (eg, included a statement or indication that the resource can be used to support one’s mental health during COVID-19);
  - Resources that gave away a free trial, subscription, or section of their app or web-based intervention during the COVID-19 pandemic.

**Table 1:** Mapped classification of digital health interventions using the Mental Health Commission of Canada (MHCC) and World Health Organization (WHO) technology classifications.

WHO classification of digital health interventions for clients	MHCC types of e-mental health technologies
Targeted client communication	App, website Instant messaging Social media Portal/electronic medical record Smartphone, wearable Artificial intelligence, big data
Untargeted client communication	App, website Instant messaging Social media Portal/electronic medical record Smartphone, wearable Artificial intelligence, big data
Client to client communication	App, website Instant messaging Social media Smartphone, wearable Artificial intelligence, big data
Personal health tracking	Portal/electronic medical record Smartphone, wearable Artificial intelligence, big data
Citizen based reporting	App, Website Instant messaging Social media Smartphone Artificial intelligence, big data
On-demand information services to clients	Search engine Artificial intelligence, big data
Telemedicine	Telehealth

### Screening Process

To calibrate the application of inclusion criteria to the identified citations in the academic literature review, 2 members of the investigator team applied the criteria independently to 30 citations ensuring an appropriate level of agreement (>80%) before further screening was conducted. The remaining citations were divided between the 2 screeners to ensure a rapid approach to citation screening. This was facilitated by an online system called Covidence [24] and was done in the following stages:

- Stage 1: citations identified from the academic database searches were uploaded into Covidence for screening;
- Stage 2: the abstracts and titles of all citations were screened utilizing the predetermined inclusion criteria. Questions regarding the eligibility of citations were discussed between the screeners, and the nominated principal applicant was consulted if a discrepancy was found;
- Stage 3: the remaining citations had the inclusion criteria applied by the screeners to the full text;
- Stage 4: preliminary data was then extracted from the articles that were included after the full-text screening was completed and entered into a data extraction table to assist with data synthesis.

### Data Synthesis

For the included academic literature, a data extraction table was created that captured basic article characteristics and results related to the research question and objectives. To identify populations that may be impacted by the digital intervention, we also collected information pertaining to the following equity-related areas: (1) Indigenous peoples and communities, (2) gender/sex, (3) race/ethnicity and culture, and (4) socioeconomic status. For apps and web-based resources, a data extraction table was created that captured basic details of the interventions (eg, target population, cost) and their specific purpose(s). Synthesis of the data from the data extraction tables was done using thematic analysis and descriptive statistics.

### Quality Assessment

We conducted a quality assessment of the included articles using the Standard Quality Assessment Criteria for Evaluating Primary Research Papers from a Variety of Fields resource (Kmet criteria) [25]. We also carried out an evaluation of the included mobile apps using the American Psychiatric Association’s App Evaluation Model [26], while leveraging the app evaluation website developed by the Division of Digital Psychiatry at the Beth Israel Deaconess Medical Center [27]. For those apps that did not have an evaluation present within the website, we carried out our own evaluation using the App Evaluation Model. This was also informed by the MHCC’s guidance on app assessment and selection [28]. Regarding the website resources, no quality assessment was performed primarily due to the volume and variety of identified website resources (eg, telemedicine, virtual peer support groups, discussion forums, etc).

## Results

### Academic Literature Findings

Results of the academic search are summarized in a PRISMA (Preferred Reporting Items for Systematic Reviews and Meta-Analyses) flow diagram shown in Multimedia Appendix 3. We found a total of 70 articles that met our inclusion criteria. While many articles specifically addressed COVID-19 (n=42, 60%), others were related to natural disasters (n=19, 27%) and human-made disasters (n=9, 14%). Most of the COVID-19–specific articles were commentaries or viewpoints (n=32, 46%). A total of 25 (36%) were primary studies and 13 (18%) were reviews. Identified articles originated primarily from the United States (n=39) but were also from China (n=6), Australia (n=4), the United Kingdom (n=4), India (n=2), Italy (n=2), New Zealand (n=2), Brazil (n=1), Canada (n=1), Croatia (n=1), Indonesia (n=1), Iraq (n=1), Ireland (n=1), Israel (n=1), the Netherlands (n=1), Pakistan (n=1), Spain (n=1), and Taiwan (n=1). A full data extraction table of all included articles is available upon request from the corresponding author.

### Gray Search Findings

We found 174 smartphone apps in our initial search. A total of 135 were identified through curated libraries, 19 via searches in the Google Play Store and Apple’s App Store, and 20 through web-based searches. Once the inclusion criteria were applied, a total of 31 mobile apps and 114 web-based resources were found. A complete list of the identified mobile apps and web-based resources can be found on the CAMH website [29].

### Identified Interventions

A list of digital interventions and their respective purposes is shown in Multimedia Appendix 4. This includes smartphone apps and web-based resources that mirror the technology categories of the WHO classification and MHCC technology typology.

### Populations That Might Benefit From or Be Disadvantaged by the Identified Digital Interventions

Most interventions impacted those with access to a device (eg, smartphone, laptop, or tablet) and an internet connection (cell phone data or wireless). Four interventions involved shifting away from in-person care to providing mental health care virtually through telemedicine platforms, videoconferencing platforms (eg, Zoom [30]), or in-house platforms during the COVID-19 pandemic [31-34]. Such interventions benefited those with a private or safe space to engage in their care [31-34]. Conversely, populations that were at a disadvantage for making use of digital interventions included those who lacked the appropriate technology or internet access (including older adults who may not be able to effectively use technology) and those living in unsafe conditions or lacking access to a private space. Additionally, most of these interventions offered regular services for existing clients, so new clients were at a disadvantage, as well as those in need of acute care.

There were 10 web-based mental health interventions focused on remote populations [35,36] and adapted for Indigenous peoples (First Nations, Inuit, and Métis) [36], victims of sexual abuse [36,37], adolescents and caregivers [38-40], and survivors of specific trauma [37,41-43]. While these advantaged a variety of populations, a few were available only in English and hence those who did not speak the language were at a disadvantage. A mobile app that focused on connection between family members and seniors [44] was available for English, Spanish, and French speakers, but, like the previously mentioned interventions, were limited to those with the know-how and technology to use it.

Two COVID-19–specific interventions—telephone and television support [45]—were advantageous for older adults and their caregivers; phone services were beneficial for individuals with substance use or opioid disorder [46]. In general, hotlines or phone-support interventions (including the three developed prior to COVID-19) [47-49] helped to expand the population to those with access to a landline or basic phone plan, including those living in transitory circumstances.

A high-risk population that emerged within our findings was veterans. Veterans were advantaged by targeted supports, including veteran-specific telemedicine programs [50], especially those who were poor, unemployed, single, or with a poor health status. Veteran-specific digital interventions such as educational and self-help resources also benefited family members [51]. A thorough list of the populations advantaged and disadvantaged by the included interventions is described in Multimedia Appendix 4.

We also collected additional information about the following equity-related areas: (1) Indigenous peoples and communities, (2) gender/sex, (3) race/ethnicity and culture, and (4) socioeconomic status. Belleville et al [36] adapted their intervention for diverse populations including members of the First Nations, Inuit, and Métis communities. Two studies addressed sex and gender considerations, recognizing that women exhibit more help-seeking behaviors [36] and were more open to sharing instances of sexual abuse [47]. Three studies addressed race/ethnicity and cultural considerations, including ones that were specifically adapted to fit a non-Western population [37], available in a variety of languages and offered translator services [47], and targeted at veterans [51]. Eleven studies considered the socioeconomic status of their end-users, providing free interventions for those who were publicly insured. Two interventions provided phone calls as an alternative to the internet [34,49], and one was tailored to older adults’ digital health literacy [45].

### What Is Known About the Effect of These Digital Interventions

Of the identified studies, 7 had information related to the effect of the intervention [36-40,45,49]. The majority (n=6) were found to be effective in reducing symptoms of posttraumatic stress disorder (PTSD), anxiety, depression, and loneliness in the various medical, natural, and man-made disaster contexts [36-40,45]. For example, one study evaluated the use of a web-based mental health intervention in tornado-affected rural and urban regions of the United States [38]. Findings from this study demonstrated that there were no significant differences in the uptake and completion of the web-based mental health intervention by rural and urban households. This suggests that digital interventions may be used to support the mental health of individuals residing in rural geographic areas, given these communities often lack in-person access to mental health services [38]. Another study underscored the effectiveness of e-therapy (electronic therapy) in treating youth with mild to moderate anxiety. An emphasis was placed on the use of digital mental health interventions within the stepped care model to improve mental health care delivery, whereby primary care physicians can have access to a menu of digital mental health interventions to recommend to parents or caregivers concerned about their child’s emotional or behavioral well-being [39,40].

Several international studies, specifically an internet-based intervention for PTSD in Iraq [37] and a community mental health relief program in Taiwan following an earthquake [49], highlighted the uptake and effect of digital mental health resources and interventions in politically and/or physically unstable regions. Individuals affected and displaced by both war or conflict and natural disasters sought mental health support through digital means and benefited from the digital support in place of access to in-person support [37,49]. In addition, a television-based intervention developed for older adults during the COVID-19 pandemic was found to be effective in reducing feelings of loneliness and distress during the isolation/quarantine period [45]. Adoption of and access to smartphones, TV devices, tablets, and computers facilitated connectedness and increased communication between older adults and their family members or caregivers. A summary of the effect for each of the identified interventions is described in Multimedia Appendix 5.

### Barriers and Facilitators to the Use of These Digital Interventions

Most included articles identified a few barriers and facilitators to the uptake and use of digital mental health interventions. These were related to the (1) technology (eg, poor connectivity, user-friendly design), (2) people using the technology (eg, knowledge about how to use the digital intervention, interest in using the intervention), and (3) context/processes in place to support or detract from the uptake of the technology (eg, a private space to use the digital intervention, organizational supports if applicable). A sample of these barriers and facilitators is shown in Textbox 1.

**Barriers and facilitators to digital mental health intervention use.**

**Barriers:**

- Difficulty using the technology
- Mistrust of the technology or security of data
- Legislation that prevents certain forms of care (eg, harm reduction)
- Lack of data sharing/interoperability
- Difficulty establishing a therapeutic alliance between people seeking care and providers due to technology-related challenges
- Poor connectivity

**Facilitators:**

- Organizational support (eg, help desk)
- Access to the technology, devices, or the software
- Access to training about the digital intervention
- Access to a specific type of mental health care
- Cost (free or provided at a limited cost)
- Ability of users to be anonymous if desired

A full list of the identified barriers and facilitators with details related to the specific type of digital intervention used is included in Multimedia Appendix 6.

## Discussion

### Principal Findings

This review identified that there are many digital interventions that could be used to support the mental health of the general Canadian population during the COVID-19 pandemic. These interventions vary in their purpose, approach, design, cost, and targeted user group. While some research and digital interventions addressed equity-related considerations, more research and focused attention should be paid to this area. Most digital interventions identified did not have any published data describing their effect within the context of COVID-19. The lack of data on the effectiveness is understandable given the timing of this review, which corresponds to the timing of the onset of the global pandemic (both in the same year) [19].

With an expanding plethora of digital mental health interventions available, with variability in cost, population focus, and type of technology used, it becomes difficult for people with lived experience of mental health problems or illnesses and providers to know which intervention to select. In addition, unawareness of the effectiveness of interventions may make clinicians wary of making specific recommendations. Fortunately, there has been some effort to support the assessment of certain types of interventions. For example the American Psychiatric Association and the MHCC both have tools to support the selection and assessment of mobile apps [26,52]. A recently created resource lists the various app libraries/curated lists, app rating tools, and a selection of digital mental health interventions, aimed at both providers and people with lived experience alike [53]. The MHCC has also developed an implementation toolkit to support organizations in their digital mental health intervention implementations [54]. Furthermore, there has been discussion regarding a role of a “digital navigator” for integrating the use of technology in practice [55].

While we identified several studies evaluating digital mental health interventions during disaster, crisis, or pandemic situations, there has been limited focus on equity factors that may influence the use and ability to obtain benefits from the intervention in all subpopulations. The equity factors specifically reviewed in this study included relevance and inclusivity of Indigenous peoples and communities, sex and gender, race/ethnicity and culture, and socioeconomic status.

One finding of this work is the lack of resources that are specific to Indigenous peoples and communities, which is a concern in the Canadian context. To ensure that the results of future works are relevant and inclusive of Indigenous peoples and communities, a few key points should be considered. Researchers and intervention developers need to ensure that Indigenous peoples are included at every stage of development and implementation [56-59]. Interventions should (1) be developed by and/or with Indigenous organizations or communities; (2) implemented and evaluated by an Indigenous organization or communities; (3) developed in accordance with OCAP (Ownership, Control, Access, and Possession) principles (these principles are specific to First Nations Peoples [60]); (4) stored appropriately and in a location that can be easily accessed by the engaged/involved Indigenous peoples or communities; and lastly, (5) all elements, including language, should ensure cultural relevance and safety. A list of resources to support this work can be found on the NunatuKavut website [61].

Other equity factors explored in this work could benefit from utilizing an appropriate framework to underpin how equity factors should be understood, examined, and evaluated. Recent work advanced by Crawford and Serhal [62] provides a Digital Health Equity Framework for how future studies may approach exploring equity within a digital landscape, emphasizing the need to consider digital health within the larger contexts of socioeconomic location of individuals and communities, which are impacted by social determinants of health.

### Strengths and Limitations

This review has several strengths and limitations. Strengths of this review included a quality appraisal of academic publications and a formal evaluation of mobile apps. Regarding limitations, this review specifically identified interventions that had been developed or modified to provide COVID-19–specific content unique to Canada. It is possible that digital mental health interventions that were developed prior to the pandemic, and without pandemic-specific content, could still be helpful. Additionally, there was great variability in the reported digital interventions, and while a quality assessment was conducted, there remains variability in the quality of the interventions available. Although we conducted several searches in a number of search engines and libraries, some digital health interventions that may meet the inclusion criteria and can support population mental health during COVID-19 might have been missed. The compiled list of interventions is not an exhaustive list of the digital mental health resources that exist to support population mental health. It presents mental health resources available to the general population and does not include those available through employment benefits programs or other workplace programs not available to the general public. In addition, the time it takes for research and publication to occur may have limited the COVID-19–specific content present in the academic literature.

### Conclusion

Numerous digital interventions that could be used to support the mental health of the general population in Canada during the COVID-19 pandemic were identified. While there are similarities to some of the nontechnology based interventions identified in a recently published review of interventions to support mental health during emergency and disaster situations [19], digital interventions present the opportunity for mental health care to be delivered at a distance. However, additional preventative measures are needed to both sustain mental wellness and to address psychological distress before severe impacts ensue. Population-based interventions are increasingly needed as a way of reducing and preventing the potential mental health impacts experienced by Canadians as a result of job loss, social isolation, changes to everyday life, both now and into the foreseeable future. To date, the interventions developed or modified to meet the current need have had minimal evaluation to determine their efficacy, as well as applicability to populations who have traditionally been disadvantaged and who continue to experience health inequities. Numerous barriers to the use of digital technology also exist for a number of individuals. Thus, a broader conversation to include concepts of equity and access is essential as these tools are developed. This rapid review demonstrates that there has been progress toward the development and adaption of digital interventions to support mental health and wellness during the COVID-19 pandemic; however, much more work needs to be done to assess the impact of these technologies.

## Appendix

Multimedia Appendix 1 Search strategy.

Multimedia Appendix 2 Gray literature search strategy.

Multimedia Appendix 3 PRISMA flow diagram.

Multimedia Appendix 4 List of digital intervention purposes and populations advantaged or disadvantaged.

Multimedia Appendix 5 Effect of digital interventions.

Multimedia Appendix 6 Barriers and facilitators to digital intervention use.

## Abbreviations

e-therapy: electronic therapy
MHCC: Mental Health Commission of Canada
OCAP: Ownership, Control, Access, and Possession
PRISMA: Preferred Reporting Items for Systematic Reviews and Meta-Analyses
PTSD: posttraumatic stress disorder
WHO: World Health Organization
