# Supplementary material for: Digital Interventions to Support Population Mental Health in Canada During the COVID-19 Pandemic: Rapid Review
Source: JMIR Ment Health. 2021 Mar 2;8(3):e26550. doi: 10.2196/26550 (PMC7927953; doi:10.2196/26550)
Supplement: Multimedia Appendix 1 [file mental_v8i3e26550_app1.docx]

**Multimedia Appendix 1: Search Strategy**

Database: Ovid MEDLINE: Epub Ahead of Print, In-Process & Other Non-Indexed Citations, Ovid MEDLINE® Daily and Ovid MEDLINE® <1946-Present>

Search Strategy:

--------------------------------------------------------------------------------

1 computer*.mp. (808086)

2 ((monitor or screen) adj4 (computer or internet or online)).mp. (3666)

3 (ehealth or e-health).mp. (6430)

4 exp telemedicine/ or videoconferencing/ (28665)

5 (teletherap* or telepsychotherap* or telepsychiatr* or telemed* or telehealth or teleconferenc* or tele-therap* or tele-psychotherap* or tele-psychiatr* or tele-med* or tele-health or tele-conferenc*).mp. (36425)

6 (eportal or e-portal or patient portal).mp. (706)

7 exp Computer Systems/ or information technology/ (177395)

8 medical records systems, computerized/ or exp electronic health records/ or health smart cards/ (38527)

9 exp Medical Informatics/ (458951)

10 (electronic health record* or electronic medical record* or EHR or clinical information system* or health information technolog*).mp. (45096)

11 (virtual reality or mobile or technology-assisted or computer-based or internet-based or information technology or web-based or technology-mediated or technology-enabled).mp. (174893)

12 virtual reality/ (1795)

13 Mobile Applications/ (5732)

14 (mobile adj2 (app or apps or therapy or therapies)).mp. (3238)

15 (mhealth or m-health).mp. (5077)

16 therapy, computer-assisted/ or diagnosis, computer-assisted/ (28551)

17 computer on wheels.mp. (6)

18 workstation on wheels.mp. (3)

19 exp Disasters/ (85291)

20 mass causa*$.mp. (22)

21 avalanche*.mp. (3109)

22 Tidal wave*.mp. (497)

23 hurricane$.mp. (3646)

24 earthquake$.mp. (9126)

25 tsunami$.mp. (2673)

26 volcan*.mp. (7209)

27 cyclo* storm$.mp. (2139)

28 tropical storm$.mp. (195)

29 flood?.mp. (9223)

30 tornado$.mp. (764)

31 landslide$.mp. (716)

32 sinkhole$.mp. (143)

33 extreme heat$.mp. (1105)

34 ash fall$.mp. (46)

35 rock fall$.mp. (17)

36 lahar?.mp. (33)

37 (severe storm$ or super storm$ or superstorm$).mp. (204)

38 heavy storm$.mp. (23)

39 disaster$.mp. (42392)

40 avalanches/ or earthquakes/ or tidal waves/ or tsunamis/ or volcanic eruptions/ or wildfires/ (6126)

41 ((((exp Coronavirus/ or exp Coronavirus Infections/ or (coronavirus* or corona virus* or OC43 or NL63 or 229E or HKU1 or HCoV* or ncov* or covid* or sars-cov* or sarscov* or Sars-coronavirus* or Severe Acute Respiratory Syndrome Coronavirus*).mp.) and ((20191* or 202*).dp. or 20190101:20301231.(ep).)) not (SARS or SARS-CoV or MERS or MERS-CoV or Middle East respiratory syndrome or camel* or dromedar* or equine or coronary or coronal or covidence* or covidien or influenza virus or HIV or bovine or calves or TGEV or feline or porcine or BCoV or PED or PEDV or PDCoV or FIPV or FCoV or SADS-CoV or canine or CCov or zoonotic or avian influenza or H1N1 or H5N1 or H5N6 or IBV or murine corona*).mp.) or ((((pneumonia or covid* or coronavirus* or corona virus* or ncov* or 2019-ncov or sars*).mp. or exp pneumonia/) and Wuhan.mp.) or (2019-ncov or ncov19 or ncov-19 or 2019-novel CoV or sars-cov2 or sars-cov-2 or sarscov2 or sarscov-2 or Sars-coronavirus2 or Sars-coronavirus-2 or SARS-like coronavirus* or coronavirus-19 or covid19 or covid-19 or covid 2019 or ((novel or new or nouveau) adj2 (CoV on nCoV or covid or coronavirus* or corona virus or Pandemi*2)) or ((covid or covid19 or covid-19) and pandemic*2) or (coronavirus* and pneumonia)).mp. or COVID-19.rx,px,ox. or severe acute respiratory syndrome coronavirus 2.os. (21119)

42 exp Acquired Immunodeficiency Syndrome/ (76222)

43 exp HIV/ (98325)

44 (Acquired adj3 Immunodeficiency adj3 Syndrome$).mp. (89813)

45 (Acquired adj3 Immuno deficiency adj3 Syndrome$).mp. (137)

46 AIDS.mp. (195159)

47 (human adj3 immunodeficiency adj3 virus).mp. (97775)

48 HIV.mp. (356989)

49 exp Hemorrhagic Fever, Ebola/ (5375)

50 ebola?.mp. (9033)

51 exp Influenza A Virus, H1N1 Subtype/ (15172)

52 H1N1.mp. (20981)

53 exp SARS Virus/ (3121)

54 exp Severe Acute Respiratory Syndrome/ (4614)

55 SARS.mp. (15252)

56 (severe adj3 acute adj3 respiratory syndrome$).mp. (12273)

57 exp Middle East Respiratory Syndrome Coronavirus/ (1063)

58 MERS.mp. (4678)

59 (middle east adj3 respiratory syndrome$).mp. (2304)

60 exp Zika Virus/ (3599)

61 exp Zika Virus Infection/ (4175)

62 Zika?.mp. (7881)

63 ((avian? or bird? or swine?) adj3 (flu? or influenza?)).mp. (16191)

64 exp Pandemics/ (9555)

65 exp Communicable Diseases, Emerging/ (5797)

66 pandemic$.mp. (35973)

67 (emerg$ adj3 disease$).mp. (20627)

68 exp disease outbreak/ (98963)

69 (disease$ adj3 outbreak$).mp. (86179)

70 exp mental health/ (37748)

71 (mental$ adj3 health$).mp. (195876)

72 (mental$ adj3 hygiene$).mp. (3290)

73 exp psychiatry/ (103722)

74 psychiatry$.mp. (93783)

75 exp mental health services/ (95002)

76 exp mental disorders/ (1231186)

77 (mental* ill* or mental* disorder* or psychiatr*).mp. [mp=title, abstract, original title, name of substance word, subject heading word, floating sub-heading word, keyword heading word, organism supplementary concept word, protocol supplementary concept word, rare disease supplementary concept word, unique identifier, synonyms] (477729)

78 depress$.mp. (540280)

79 ptsd?.mp. (24784)

80 (trauma$ adj3 disorder$).mp. (45308)

81 or/1-18 [digital intervention set] (1294371)

82 or/19-40 [disasters set] (117133)

83 or/41-69 [pandemic/disease outbreak set] (650778)

84 or/70-80 [mental health set] (1845400)

85 81 and (82 or 83) and 84 (3136)

86 exp animal/ not exp human/ (4705014)

87 86 not 87 (3180)

TOTAL WHEN LIMITED TO 2002-CURRENT (2667)
