# Supplementary material for: Digital Interventions to Support Population Mental Health in Canada During the COVID-19 Pandemic: Rapid Review
Source: JMIR Ment Health. 2021 Mar 2;8(3):e26550. doi: 10.2196/26550 (PMC7927953; doi:10.2196/26550)
Supplement: Multimedia Appendix 2 [file mental_v8i3e26550_app2.docx]

**Multimedia Appendix 2: Grey Literature Search Strategy**

**Google Searches**

Searches on Apps or Web based interventions to promote mental health

COVID-19/ apps/Mental Health

- Search string: covid OR covid-19 OR coronavirus OR  "corona virus"  apps "mental health"

COVID-19/ web-based/Mental Health

- Search string: covid OR covid-19 OR coronavirus OR  "corona virus"  “web based” "mental health"

**Million Short Searches**

Filters:

- Results limited to Australia, Canada, US and UK
- Removal of the top 1000, the top 10k, the top 100k and top million hits

Search strings:

- Mental Health COVID 19 APPS
- Mental Health COVID 19 Web-Based
