# Supplementary material for: Digital Interventions to Support Population Mental Health in Canada During the COVID-19 Pandemic: Rapid Review
Source: JMIR Ment Health. 2021 Mar 2;8(3):e26550. doi: 10.2196/26550 (PMC7927953; doi:10.2196/26550)
Supplement: Multimedia Appendix 3 [file mental_v8i3e26550_app3.docx]

**Multimedia Appendix 3: PRISMA Flow Diagram**

Records excluded
(n =2664)

Records screened
(n =2907)

Full-text articles excluded
(n = 173)

**Identification**

**Eligibility**

**Included**

**Screening**

Duplicates excluded
(n =2907)

Full-text articles assessed for eligibility
(n =243)

Records identified through database searching
(n = 3772)

Included in synthesis
(n =70)
