# Supplementary material for: Digital Interventions to Support Population Mental Health in Canada During the COVID-19 Pandemic: Rapid Review
Source: JMIR Ment Health. 2021 Mar 2;8(3):e26550. doi: 10.2196/26550 (PMC7927953; doi:10.2196/26550)
Supplement: Multimedia Appendix 4 [file mental_v8i3e26550_app4.docx]

**Multimedia Appendix 4: List of digital intervention purposes and populations advantaged/disadvantaged**

| **Citation** | **Technology & Purpose** | **Populations Advantaged** | **Populations Disadvantaged** |
| --- | --- | --- | --- |
| Barney, A., Buckelew, S., Mesheriakova, V., & Raymond-Flesch, M. (2020). The COVID-19 pandemic and rapid implementation of adolescent and young adult telemedicine: challenges and opportunities for innovation. *Journal of Adolescent Health*. | **Telemedicine:** As a result of COVID-19 a clinic has shifted away from in-person visits to virtual care through telemedicine. | - Individuals who have access to technology and internet - Individuals who have a private and safe space | - Individuals who lack internet or technology access - Individuals who do not have access to a private space - Individuals living in unsafe conditions |
| Belleville, G., Lebel, J., Ouellet, M. C., Békés, V., Morin, C. M., Bergeron, N., & Macmaster, F. P. (2019). Resilient-An online multi-dimensional treatment to promote resilience and better sleep: a randomized controlled trial. *Sleep Medicine*, *64*, S214-S215. | **Online Platform:** Web-based self-help CBT intervention for PTSD resilience. | - Remote/rural populations affected by the Fort McMurrary Fires - Adapted for Indigenous Peoples and victims of sexual abuse. | N/A |
| Bunnell, B. E., Davidson, T. M., Dewey, D., Price, M., & Ruggiero, K. J. (2017). Rural and urban/suburban families' use of a web-based mental health intervention. *Telemedicine and e-Health*, *23*(5), 390-396. | **Online Platform:** Web-based disaster mental health intervention for adolescents and caregivers. | - Rural and urban populations - Adolescents and caregivers - Individuals who have access to technology and internet - English Speaking | - Individuals who lack internet or technology access - Non-English speaking |
| Bush, N. E., Bosmajian, C. P., Fairall, J. M., McCann, R. A., & Ciulla, R. P. (2011). afterdeployment. org: A web-based multimedia wellness resource for the postdeployment military community. *Professional Psychology: Research and Practice*, *42*(6), 455. | **Online Platform**: Provides veterans with access to educational and self-help resources post deployment. | - Veterans, family members of military personnel | N/A |
| Der-Martirosian, C., Chu, K., & Dobalian, A. (2020). Use of Telehealth to Improve Access to Care at the United States Department of Veterans Affairs During the 2017 Atlantic Hurricane Season. *Disaster medicine and public health preparedness*, 1-5. | **Telemedicine:** Providing veterans with telehealth services when in-person care is not feasible | - Veterans (typically who are poor, unemployed, single, poor health status) | N/A |
| Goodman-Casanova, J. M., Dura-Perez, E., Guzman-Parra, J., Cuesta-Vargas, A., & Mayoral-Cleries, F. (2020). Telehealth home support during COVID-19 confinement for community-dwelling older adults with mild cognitive impairment or mild dementia: survey study. *Journal of Medical Internet Research*, *22*(5), e19434. | **Telemedicine:** Television and telephone-based health social support for older adults during the COVID-19 pandemic | - Older adults with mild cognitive impairments, and/or mild dementia - Caregivers and family members | - Individuals who lack access to telephone communication or cable TV. |
| Lee, I. H., Chen, C. C., Yeh, T. L., Chen, K. C., Lee, C. K., Chen, P. S., ... & Lu, R. B. (2010). A community mental health survey and relief program in Taiwan after the great earthquake—Implementation, clinical observation and evaluation. *Stress and health*, *26*(4), 269-279. | **Telemedicine:** A two-programme mental health program was established after an earthquake, phone support and a mobile clinic. | - Non-treatment seeking, high-risk survivors of the earthquake - Individuals with psychiatric comorbidities | N/A |
| Looi, J. C., & Pring, W. (2020). Private metropolitan telepsychiatry in Australia during Covid-19: current practice and future developments. *Australasian Psychiatry*, 1039856220930675. | **Telemedicine:** Telephone and video-consultations (tele-psychiatry) were deployed to replace in-person care as a result of COVID-19. | - Most effective for current or previously known patients - Individuals who have access to a private or safe space | - Individuals with acute psychiatric presentations - New patients - Individuals who do not have access to a private or safe space |
| Mack, D., Brantley, K. M., & Bell, K. G. (2007). Mitigating the health effects of disasters for medically underserved populations: electronic health records, telemedicine, research, screening, and surveillance. *Journal of Health Care for the Poor and Underserved*, *18*(2), 432-442. | **Telemedicine:** Delivery of virtual care for underserved communities after Hurricane’s Rita and Katrina | - Individuals who reside in underserved communities where there is limited access to mental health care providers. | N/A |
| Meinert, E., Milne-Ives, M., Surodina, S., & Lam, C. (2020). Agile requirements engineering and software planning for a digital health platform to engage the effects of isolation caused by social distancing: case study. *JMIR Public Health and Surveillance*, *6*(2), e19297. | **Mobile App:** ADAPT-CAFÉ is an app that assists family members in maintaining contact with seniors. Purpose of the app is to reduce loneliness during the isolation period. | - Older adults, family members and caregivers - English, Spanish and French Speakers - Individuals who have a smart device and internet access | Individuals who do not have a smart device and internet access |
| Moor, S., Williman, J., Drummond, S., Fulton, C., Mayes, W., Ward, N., ... & Stasiak, K. (2019). ‘E’therapy in the community: Examination of the uptake and effectiveness of BRAVE (a self-help computer programme for anxiety in children and adolescents) in primary care. *Internet Interventions*, *18*, 100249. | **Online Platform:** Web-based therapy assisted program for children and adolescents. | - Children and adolescents with mild-moderate anxiety - Children with access to technology and internet | - Children/adolescents with depression, self-harming behaviour or severe anxiety - Children without access to technology and internet |
| Olwill, C., Mc Nally, D., & Douglas, L. (2020). Psychiatrist experience of remote consultations by telephone in an outpatient psychiatric department during the COVID-19 pandemic. *Irish Journal of Psychological Medicine*, 1-8. | **Telemedicine:** The use of telephone psychiatric consultations during COVID-19 | - Pre-existing patients or patients with existing relationships with the physicians - Technology access | - New patients - Those with limited English fluency were more likely to have issues using the technology - Individuals with cognitive impairments had more issues using the technology |
| Price, M., Davidson, T. M., Andrews, J. O., & Ruggiero, K. J. (2013). Access, use and completion of a brief disaster mental health intervention among Hispanics, African-Americans and Whites affected by Hurricane Ike. *Journal of telemedicine and telecare*, *19*(2), 70-74. | **Online Platform:** Disaster recovery mental health platform for individuals after Hurricane Ike. | - English Speaking - Individuals with internet access and access to a landline | - Individuals who lack access to internet or a landline - Non-English speakers |
| Price, M., Gros, D. F., McCauley, J. L., Gros, K. S., & Ruggiero, K. J. (2012). Nonuse and dropout attrition for a web-based mental health intervention delivered in a post-disaster context. *Psychiatry: Interpersonal & Biological Processes*, *75*(3), 267-284. | **Online Platform:** Disaster recovery mental health platform for individuals after Hurricane Ike. | - Individuals with internet access and access to a landline - English Speaking | - Individuals who lack access to internet or a landline - Non-English speakers |
| Price, M., Yuen, E. K., Davidson, T. M., Hubel, G., & Ruggiero, K. J. (2015). Access and completion of a web-based treatment in a population-based sample of tornado-affected adolescents. *Psychological services*, *12*(3), 283. | **Online Platform:** Bounce Back Now – an intervention for adolescents living in tornado affected communities | - Adolescents and their family members - Individuals with internet access and access to a landline - English Speaking | - Adolescents residing in institutional settings - Individuals who lack access to internet or a landline - Non-English speakers |
| Reifels, L., Bassilios, B., & Pirkis, J. (2012). National telemental health responses to a major bushfire disaster. *Journal of telemedicine and telecare*, *18*(4), 226-230. | **Telemedicine:** telemental health services through phone-based counselling in response to the Victoria, Australia Bush Fires | - Free of cost - Phone service allows for confidentiality and enhanced privacy - Accessible to individuals in rural and urban settings | - Populations where infrastructure (phone lines) was destroyed - Populations who did not have phone access/service - Populations living in transitory circumstances |
| Ruggiero, K. J., Price, M., Adams, Z., Stauffacher, K., McCauley, J., Danielson, C. K., ... & Carpenter, M. J. (2015). Web intervention for adolescents affected by disaster: Population-based randomized controlled trial. *Journal of the American Academy of Child & Adolescent Psychiatry*, *54*(9), 709-717. | **Online Platform:** Bounce Back now was assessed for use in a post-disaster context (Tornado) | - Parents and adolescents affected by the Tornados - Individuals with internet or technology access | - Individuals without internet or technology access - Individuals residing in shelters |
| Ruggiero, K. J., Resnick, H. S., Acierno, R., Coffey, S. F., Carpenter, M. J., Ruscio, A. M., ... & Bucuvalas, M. (2006). Internet-based intervention for mental health and substance use problems in disaster-affected populations: a pilot feasibility study. *Behavior therapy*, *37*(2), 190-205. | **Online Platform**: An internet-based intervention to support mental health after the 9/11 attacks. | - Adults traumatized by the attacks - English Speakers | - Populations who lacked internet access or technology access - Non-English speakers - Populations excluded were more likely to be women, black and Latino/a people, unemployed, older adults, less educated. |
| Ruggiero, K. J., Resnick, H. S., Paul, L. A., Gros, K., McCauley, J. L., Acierno, R., ... & Galea, S. (2012). Randomized controlled trial of an internet-based intervention using random-digit-dial recruitment: the Disaster Recovery Web project. *Contemporary clinical trials*, *33*(1), 237-246. | **Online Platform**: Web-based intervention to support mental health after Hurricane Ike | - Individuals with internet access and access to technology - English Speaking | - Populations who lacked internet access or technology access - Non-English speakers - Populations residing in institutionalized settings |
| Samuels, E. A., Clark, S. A., Wunsch, C., Keeler, L. A. J., Reddy, N., Vanjani, R., & Wightman, R. S. (2020). Innovation during COVID-19: Improving addiction treatment access. *Journal of Addiction Medicine*. | **Telemedicine:** Phone hotline “tele-bridge” for individuals with SUD | - Individuals with SUD/Opioid use disorder | - N/A |
| van Agteren, J., Bartholomaeus, J., Fassnacht, D. B., Iasiello, M., Ali, K., Lo, L., & Kyrios, M. (2020). Using Internet-Based Psychological Measurement to Capture the Deteriorating Community Mental Health Profile During COVID-19: Observational Study. *JMIR Mental Health*, *7*(6), e20696. | **Online Platform:** Internet-based self assessment tool to monitor mental well being and resilience during COVID-19 | - Free of cost - Individuals with internet access and technology access | - Populations who lacked internet access or technology access |
| Wagner, B., Schulz, W., & Knaevelsrud, C. (2012). Efficacy of an Internet-based intervention for posttraumatic stress disorder in Iraq: a pilot study. *Psychiatry research*, *195*(1-2), 85-88. | **Online Platform:** Internet-based psychological intervention to treat Iraq citizens with PTSD. | - Patients with PTSD - Populations/Cultures in which mental health is heavily stigmatized – the platform allowed for a level of anonymity - Women (many participants were victims of sexual assault and due to the stigma surrounding sexual assault, they were hesitant to seek help. This platform allowed for them to seek help for their PTSD symptoms. | - Individuals with limited to no access to stable internet |
| Wunsch-Hitzig, R., Plapinger, J., Draper, J., & Del Campo, E. (2002). Calls for help after September 11: a community mental health hot line. *Journal of urban health*, *79*(3), 417-428. | **Telemedicine:** Community mental health phone hotline to support New Yorkers post 9/11. | - General population (with and without a diagnosed mental health disorder) - Individuals who speak English and those who do not (translation services available) | - Older adults – not many seniors used the phoneline – there is evidence that seniors are less likely to reach out for help during a disaster. |
| Yellowlees, P., Nakagawa, K., Pakyurek, M., Hanson, A., Elder, J., & Kales, H. C. (2020). Rapid Conversion of an Outpatient Psychiatric Clinic to a 100% Virtual Telepsychiatry Clinic in Response to COVID-19. *Psychiatric Services*, appi-ps. | **Telemedicine:** Use of EPIC MyChart and/or Zoom to conduct virtual care consultations. Phone consultations were also conducted. | - Individuals with internet access and technology access | - Older Adults - Populations who lacked internet access or technology access |
