# Supplementary material for: Digital Interventions to Support Population Mental Health in Canada During the COVID-19 Pandemic: Rapid Review
Source: JMIR Ment Health. 2021 Mar 2;8(3):e26550. doi: 10.2196/26550 (PMC7927953; doi:10.2196/26550)
Supplement: Multimedia Appendix 5 [file mental_v8i3e26550_app5.docx]

**Multimedia Appendix 5: Effect of Digital Interventions**

| **Citation** | **Efficacy of the Intervention** |
| --- | --- |
| Belleville, G., Lebel, J., Ouellet, M. C., Békés, V., Morin, C. M., Bergeron, N., & Macmaster, F. P. (2019). Resilient-An online multi-dimensional treatment to promote resilience and better sleep: a randomized controlled trial. *Sleep Medicine*, *64*, S214-S215. | - The intervention was found to reduce symptoms of PTSD, insomnia and depression in victims/evacuees from the 2016 Fort McMurray Wildfires |
| Bunnell, B. E., Davidson, T. M., Dewey, D., Price, M., & Ruggiero, K. J. (2017). Rural and urban/suburban families' use of a web-based mental health intervention. *Telemedicine and e-Health*, *23*(5), 390-396. | - There were no significant differences in the completion rate of the intervention regardless of geographic location or generation, indicating that rural families (adolescents and caregivers) completed and used the web-based intervention similarly to the urban/suburban families. - These findings prove the efficacy of technology-based resources in rural populations, particularly younger generations, in supporting their mental health - Rural residents are typically underserved or lack access to mental health care services; this study proves that web-based mental health interventions can be used to support rural populations. |
| Goodman-Casanova, J. M., Dura-Perez, E., Guzman-Parra, J., Cuesta-Vargas, A., & Mayoral-Cleries, F. (2020). Telehealth home support during COVID-19 confinement for community-dwelling older adults with mild cognitive impairment or mild dementia: survey study. *Journal of Medical Internet Research*, *22*(5), e19434. | - Access to smartphones, computers, tablets and TV-based devices facilitates connectedness and communication, and reduces feelings of isolation - As social isolation and loneliness are associated with poorer cognition, special attention must be drawn to people with mild cognitive impairment or mild dementia in regard to social activities during confinement. The set-up of phone support lines has been found to be effective in providing health and social support to this population during times of isolation. |
| Lee, I. H., Chen, C. C., Yeh, T. L., Chen, K. C., Lee, C. K., Chen, P. S., ... & Lu, R. B. (2010). A community mental health survey and relief program in Taiwan after the great earthquake—Implementation, clinical observation and evaluation. *Stress and health*, *26*(4), 269-279. | - Results suggest that the number of victims following a disaster who seek mental health support can be boosted by the implementation of an online service and aid-referral system. As well, the implementation of a mobile clinic that integrates local resources and non-professional support with professional mental health care can increase the efficiency of mental health care delivery. |
| Moor, S., Williman, J., Drummond, S., Fulton, C., Mayes, W., Ward, N., ... & Stasiak, K. (2019). ‘E’therapy in the community: Examination of the uptake and effectiveness of BRAVE (a self-help computer programme for anxiety in children and adolescents) in primary care. *Internet Interventions*, *18*, 100249. | - The tool was found to be an effective e-therapy tool in treating children with mild-moderate anxiety. This adds to the importance of having a menu of tools for primary care workers to utilize when families/parents contact them with concerns about their children’s emotional and behavioural issues. - E-therapies are proving to be an important part of the stepped-care model for improving public mental health |
| Ruggiero, K. J., Price, M., Adams, Z., Stauffacher, K., McCauley, J., Danielson, C. K., ... & Carpenter, M. J. (2015). Web intervention for adolescents affected by disaster: Population-based randomized controlled trial. *Journal of the American Academy of Child & Adolescent Psychiatry*, *54*(9), 709-717. | - A web-based approach has the potential for meaningful penetration post-disasters. Such tools may be helpful in preventing escalation of symptoms for at-risk youth and adolescents. It also supports resource allocation but identifying the youth at highest risk and should be directed to more intensive levels of care. |
| Wagner, B., Schulz, W., & Knaevelsrud, C. (2012). Efficacy of an Internet-based intervention for posttraumatic stress disorder in Iraq: a pilot study. *Psychiatry research*, *195*(1-2), 85-88. | - Participants reported reduced PTSD, intrusion, avoidance, depression and anxiety symptoms after use of the intervention - Findings prove that even when living in difficult living conditions, people with PTSD seek and benefit from psychological treatment provided entirely through the internet. The programs uptake confirms that new technologies can be used to provide humanitarian aid in the form of e-mental health services, even in highly unstable areas. |
