# Supplementary material for: Digital Interventions to Support Population Mental Health in Canada During the COVID-19 Pandemic: Rapid Review
Source: JMIR Ment Health. 2021 Mar 2;8(3):e26550. doi: 10.2196/26550 (PMC7927953; doi:10.2196/26550)
Supplement: Multimedia Appendix 6 [file mental_v8i3e26550_app6.docx]

**Multimedia Appendix 6: Barriers/Facilitators to Digital Intervention Use**

| **Citation** | **Facilitators to Use** | **Barriers to Use** |
| --- | --- | --- |
| Barney, A., Buckelew, S., Mesheriakova, V., & Raymond-Flesch, M. (2020). The COVID-19 pandemic and rapid implementation of adolescent and young adult telemedicine: challenges and opportunities for innovation. *Journal of Adolescent Health*. | - Institutional support for telemedicine - Access to technology and training for providers and clerical staff to adequately adjust to changes in care delivery - Modification to payment models for services | - Patient and provider acceptance of technology - Technical connectivity challenges - Infrastructure to support HIPAA compliance - Deviations from clinical standards of care |
| Bunnell, B. E., Davidson, T. M., Dewey, D., Price, M., & Ruggiero, K. J. (2017). Rural and urban/suburban families' use of a web-based mental health intervention. *Telemedicine and e-Health*, *23*(5), 390-396. | N/A | - Limited education surrounding the benefits of digital mental health services/care (stigma & psychoeducation) |
| Der-Martirosian, C., Chu, K., & Dobalian, A. (2020). Use of Telehealth to Improve Access to Care at the United States Department of Veterans Affairs During the 2017 Atlantic Hurricane Season. *Disaster medicine and public health preparedness*, 1-5. | - Improves access to care for those who may not have a usual source of healthcare | - Lack of critical electrical infrastructure to support telehealth services, especially following natural disasters |
| Der-Martirosian, C., Griffin, A. R., Chu, K., & Dobalian, A. (2019). Telehealth at the US Department of Veterans Affairs after Hurricane Sandy. *Journal of telemedicine and telecare*, *25*(5), 310-317. | - Recent advances in technology have simplified disaster communication, making telemedicine more accessible to a number of hospitals and healthcare systems. | - It is still challenging to effectively implement telehealth programmes within integrated healthcare systems especially in the context of changes to care delivery. |
| Olwill, C., Mc Nally, D., & Douglas, L. (2020). Psychiatrist experience of remote consultations by telephone in an outpatient psychiatric department during the COVID-19 pandemic. *Irish Journal of Psychological Medicine*, 1-8. | - Increased flexibility and convenience in having virtual consultations | - Therapeutic Alliance affected by phone consultations. Difficulties in establishing a rapport and trust with patients is a barrier to the use of telepsychiatry - Undergraduate and medical school training did not teach, or prepare the physicians for phone consultations |
| Price, M., Davidson, T. M., Andrews, J. O., & Ruggiero, K. J. (2013). Access, use and completion of a brief disaster mental health intervention among Hispanics, African-Americans and Whites affected by Hurricane Ike. *Journal of telemedicine and telecare*, *19*(2), 70-74. | - Web-based approaches address several predisposing factors that prevent mental health care access, including reducing stigma and allowing participants for greater autonomy in their care journey | - N/A |
| Price, M., Gros, D. F., McCauley, J. L., Gros, K. S., & Ruggiero, K. J. (2012). Nonuse and dropout attrition for a web-based mental health intervention delivered in a post-disaster context. *Psychiatry: Interpersonal & Biological Processes*, *75*(3), 267-284. | - Allows for flexibility and reduces travel, expenses, privacy and stigma implications | - N/A |
| Reifels, L., Bassilios, B., & Pirkis, J. (2012). National telemental health responses to a major bushfire disaster. *Journal of telemedicine and telecare*, *18*(4), 226-230. | - Free services, with quick and reliable responses, less confrontational medium which allows for great privacy and confidentiality - Use of existing platforms and reputable service providers and telephone numbers - Collaboration between federal, provincial and municipal disaster and mental health services | - Destroyed infrastructure as a result of the natural disaster, disconnected phone lines, out of range mobile phones, high mobile phone costs, lack of information on the available mental health supports - Lack of data sharing between jurisdictions - limited the ability for providers to learn about the emerging patterns of service demand |
| Samuels, E. A., Clark, S. A., Wunsch, C., Keeler, L. A. J., Reddy, N., Vanjani, R., & Wightman, R. S. (2020). Innovation during COVID-19: Improving addiction treatment access. *Journal of Addiction Medicine*. | N/A | - Sustainability of using telehealth for the substance use disorder buprenorphine initiation relies heavily on legislation and regulatory challenges. The ryan height online pharmacy consumer protection act prohibits the prescription of controlled substances without an initial in-person visit with a provider. |
| Wagner, B., Schulz, W., & Knaevelsrud, C. (2012). Efficacy of an Internet-based intervention for posttraumatic stress disorder in Iraq: a pilot study. *Psychiatry research*, *195*(1-2), 85-88. | - Offers, in particular, women the ability to receive therapeutic treatment for PTSD in relative anonymity - The general anonymity of the program was a facilitator for use by the population – especially due to the highly stigmatized nature of mental health. | - Distrust in the platform/program (a number of users stopped using the platform as they suspected it was supported by foreign services such as the CIA or Mossad) |
| Yellowlees, P., Nakagawa, K., Pakyurek, M., Hanson, A., Elder, J., & Kales, H. C. (2020). Rapid Conversion of an Outpatient Psychiatric Clinic to a 100% Virtual Telepsychiatry Clinic in Response to COVID-19. *Psychiatric Services*, appi-ps. | - N/A | - Inability for patients to use technology or lacking sufficient technology hardware |
